# Treatment With Adalimumab 80 mg Every Other Week in Inflammatory Bowel Disease: Results of Treatment Intensification in Clinical Practice

**DOI:** 10.1093/crocol/otac051

**Published:** 2023-01-10

**Authors:** Marta Calvo Moya, Yago González Lama, Belén Ruíz Antorán, Ignacio Omella Usieto, Ismael el Hajra Martinez, Elena Santos Pérez, Belén Menchén Viso, Virginia Matallana Royo, Irene González Partida, Rocío de Lucas Tellez de Meneses, Pablo Bella Castillo, Macarena González Rodriguez, María Isabel Vera Mendoza

**Affiliations:** Department of Gastroenterology and Hepatology, Hospital Universitario Puerta de Hierro Majadahonda, Madrid, Spain; Department of Gastroenterology and Hepatology, Hospital Universitario Puerta de Hierro Majadahonda, Madrid, Spain; Clinical Pharmacology Department, Hospital Universitario Puerta de Hierro Majadahonda, Madrid, Spain; Department of Gastroenterology and Hepatology, Hospital Universitario Puerta de Hierro Majadahonda, Madrid, Spain; Department of Gastroenterology and Hepatology, Hospital Universitario Puerta de Hierro Majadahonda, Madrid, Spain; Clinical Pharmacology Department, Hospital Universitario Puerta de Hierro Majadahonda, Madrid, Spain; Hospital Pharmacy Service, Hospital Universitario Puerta de Hierro Majadahonda, Madrid, Spain; Department of Gastroenterology and Hepatology, Hospital Universitario Puerta de Hierro Majadahonda, Madrid, Spain; Department of Gastroenterology and Hepatology, Hospital Universitario Puerta de Hierro Majadahonda, Madrid, Spain; Department of Gastroenterology and Hepatology, Hospital Universitario Puerta de Hierro Majadahonda, Madrid, Spain; Department of Gastroenterology and Hepatology, Hospital Universitario Puerta de Hierro Majadahonda, Madrid, Spain; Department of Gastroenterology and Hepatology, Hospital Universitario Puerta de Hierro Majadahonda, Madrid, Spain; Department of Gastroenterology and Hepatology, Hospital Universitario Puerta de Hierro Majadahonda, Madrid, Spain

**Keywords:** adalimumab, inflammatory bowel disease, treatment intensification, 80 mg every other week, clinical remission

## Abstract

**Background:**

Loss of response to anti-tumor necrosis factor drugs in patients with inflammatory bowel disease (IBD) is frequent and, in case of low drug levels, treatment intensification is recommended. In addition, in cases in which clinical response without attainment of remission (clinical, endoscopic, or radiological), intensification could be justified since higher drug levels are associated with better outcomes. For adalimumab (ADA), the standard intensification regimen is 40 mg every week (ew). Availability of ADA 80 mg prefilled pens has enabled every other week (eow) intensification. We assessed the clinical efficacy of intensification with ADA 80 mg eow.

**Methods:**

This retrospective study was conducted at a tertiary hospital in Spain. Patients with IBD receiving maintenance ADA 80 mg eow with clinical, biomarker, and drug-level assessments were included. Demographics and clinical, biological, and endoscopic evaluation of the disease before and after ADA intensification, and pharmacokinetic assessments, were collected.

**Results:**

Eighty-seven patients (72 Crohn’s disease, 15 ulcerative colitis; average age 50 years) were included. Reasons for ADA intensification were: low ADA levels—<5 µg mL^−1^—(17%), low ADA levels—<5 µg mL^−1^—without clinical response (63%), clinical response without clinical remission (15%) and active disease on objective evaluation (including colonoscopy, magnetic resonance imaging, capsule endoscopy, and/or intestinal ultrasound; 5%). Following treatment intensification to ADA 80 mg eow, 75 patients (86%) were in clinical remission and 69 (79.3%) were in biologic remission (clinical remission and normalization of biomarkers). After a median follow-up of 19 months (interquartile range 13–25), 63 patients (72%) remained on treatment and in clinical remission. There were no serious infections, hospitalizations, or deaths. Drug costs did not increase with the 80 mg eow regimen versus a standard intensification regimen.

**Conclusions:**

ADA intensification to 80 mg eow was safe, effective, and did not increase drug costs versus standard intensification to 40 mg ew in our experience.

## Graphical Abstract

**Graphical Abstract:**
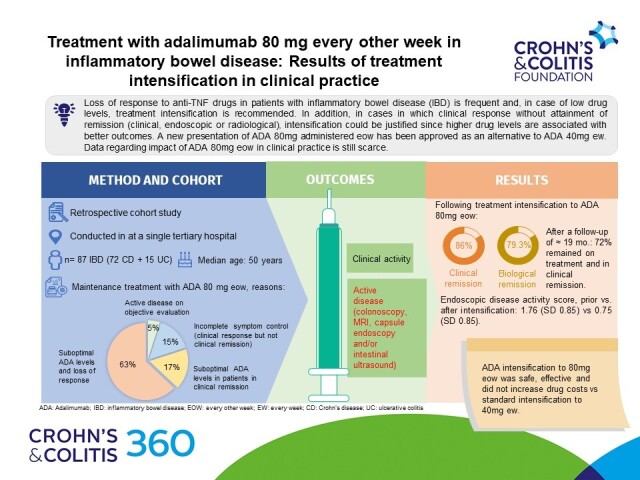

## Introduction

Inflammatory bowel disease (IBD), comprising ulcerative colitis (UC) and Crohn’s disease (CD), is characterized by chronic and relapsing inflammation of the gastrointestinal tract that can result in progressive and irreversible bowel damage.^1–3^ Genetic susceptibility, environmental factors, and aberrant immune responses are thought to contribute to the complex pathogenesis of IBD.^3^ Pathophysiology of IBD is based on an uncontrolled inflammatory process, and immunomodulator agents are the cornerstone of IBD treatment.^1,2^ Introduction of biologic agents such as anti-tumor necrosis factor (TNF) drugs, revolutionized the management of IBD through targeting the biological pathways underlying the inflammatory processes that drive these diseases.^1,2^ However, loss of response to anti-TNF drugs or clinical response without attainment of remission (clinical, radiological, or endoscopic) in patients with IBD is not uncommon. It is estimated that approximately 30% of patients who respond to induction therapy lose response during the first year of treatment, with an additional 13% of patients losing response in the following years.^4–6^

Current guidelines for the treatment of UC and CD recommend switching therapy to an alternative biologic agent in case of treatment failure with an anti-TNF agent.^1,2^ However, a proactive approach has also been proposed using therapeutic drug monitoring to determine plasma drug levels in patients with loss of response. Using this approach, a loss of response to anti-TNF drugs in the context of low plasma drug levels, can be managed initially through treatment intensification.^7–10^ In this sense, according to the treat-to-target strategy proposed in the STRIDE-I consensus^11^ and recently revised in the STRIDE-II consensus,^12^ intensification could also be justified to achieve more ambitious goals than symptom remission, such as endoscopic remission (mucosal healing), since they are associated with improved long-term results. In the case of the anti-TNF agent adalimumab (ADA), the standard intensification regimen is 40 mg every week (ew). This strategy has shown to be safe and effective with 80% of patients achieving clinical response at 3 months and 61% achieving clinical response at 12 months.^13^ In this retrospective observational study, the standard intensification regimen was associated with a significant increase in costs.^1,2^

In recent years, an ADA 80 mg prefilled pen has been marketed. Although its every other week (eow) administration is particularly attractive in cases where treatment optimization is required in IBD patients with inadequate or loss of response to the standard 40 mg ew ADA regimen, available published experience is scarce. Open-label studies in IBD have reported similar pharmacokinetics to ADA 40 mg ew,^14^ with an efficacy and safety profile consistent with the already known safety profile of ADA.^14,15^ Furthermore, dose optimization of ADA to 80 mg eow improved control of disease activity in patients with CD who had lost response to maintenance ADA 40 mg ew.^15^ This pharmacokinetic, efficacy, and safety profile has been reproduced in case series, albeit with a limited number of patients, and during short-term follow-up.^16–20^

Here, we report the results of a retrospective evaluation of clinical, endoscopic, and radiological outcomes for a cohort of patients with IBD who underwent ADA treatment intensification to 80 mg eow to assess the efficacy in terms of clinical remission in real-world clinical practice. Secondary objectives were to assess the efficacy of treatment intensification with ADA 80 mg eow in terms of biological remission, changes in biochemical parameters of activity and ADA levels after intensification, as well as the identification of factors associated with treatment discontinuation.

## Materials and Methods

This was a retrospective cohort study conducted in at a single tertiary hospital in Spain.

### Patients

The study included all consecutive patients with IBD receiving maintenance treatment with ADA 80 mg eow who had clinical, biomarker, and drug-level assessments from January 2018 to December 2020. To identify patients for ADA treatment intensification we utilized an ADA level of <5 µg mL^−1^ as an appropriate indicator of subtherapeutic plasma levels.

Patient characteristics were collated from the medical records and included sex, age, smoking status, age at diagnosis, type of IBD (CD, UC, IBD unclassified), location, disease behavior (inflammatory, stenosing, or fistulising), perianal disease, extraintestinal manifestations, previous surgery for IBD, concurrent use of immunomodulators, previous treatments for IBD, starting date of ADA, response to ADA, date of loss of response (when it occurred), starting date of ADA intensification, response to escalated ADA dose, and adverse events with escalated treatment. Additional parameters such as endoscopic and/or radiological evaluation (intestinal ultrasound or magnetic resonance imaging [MRI]) were assessed before and after intensification when available.

### Outcomes

Disease activity data were obtained retrospectively from medical records based on ICD-10 codes, from pharmacy databases (Farmatools) in which the search was filtered by drug, from radiology by diagnosis (ENDOBASE, Olimpus, for intestinal ultrasound; Syngo Imaging, Siemens, for MRI) and from endoscopies (ENDOBASE, Olimpus).

Disease activity was assessed in a number of ways. Clinical disease activity was assessed using the Harvey–Bradshaw Index (HBI) for CD patients and the Partial Mayo Score (PMS) for UC patients. Endoscopic disease activity was scored using the Simple Endoscopic Score for Crohn’s Disease (SES-CD) for luminal CD, the Rutgeerts Index for postsurgical recurrence of CD, and the Mayo Endoscopic Score for UC as follows:

- 0: No activity (SES-CD 0–3; Mayo Endoscopic Score 0; Rutgeerts Index i0)
- 1: Mild activity (SES-CD 4–10; Mayo Endoscopic Score 1; Rutgeerts Index i1)
- 2: Moderate activity (SES-CD 11–20; Mayo Endoscopic Score 2; Rutgeerts Index i2)
- 3: Severe activity (SES-CD >20; Mayo Endoscopic Score 3; Rutgeerts Index i3–4)

Radiological disease activity was assessed by measuring the thickness, mesenteric fat enhancement, and blood flow at the level of the intestinal wall by MRI and/or intestinal ultrasound. Biological disease activity was determined on the basis of clinical response and levels of fecal calprotectin (FC; feces) and C-reactive protein (CRP; peripheral blood).

Clinical remission was defined as an HBI score ≤4 for CD patients and a PMS score ≤2 for UC patients. Endoscopic remission was defined as an SES-CD score ≤2, a Rutgeerts Index score of i0, or a Mayo Endoscopic Score of 0. Radiological remission was defined as complete normalization of inflammatory parameters on imaging. Biological remission was defined as clinical remission and normalization of biomarkers (FC <250 µg g^−1^ and CRP <5 mg dL^−1^).

Clinical response was defined as a decrease in HBI score of ≥3 points or in Mayo Endoscopic Score of ≥3 points from baseline.^21^ Endoscopic response was defined as a decrease in the SES-CD score of >50% from baseline, a decrease in the Rutgeerts Index score ≤i2 with at least 1 point decrease from baseline and a decrease in the Mayo Endoscopic Score ≥1 from baseline. Radiological response was defined as a decreased thickness of the intestinal wall, decreased mesenteric fat enhancement, and decreased blood flow at the level of the intestinal wall compared with baseline.

The economic impact of treatment intensification at our center was considered as the cost price of ADA pens (2 × 40 mg dose pens, one 80 mg dose pen). The price was calculated on the basis of the cost rate of both pens according to the data provided by our pharmacy service.

### Pharmacokinetics

Pharmacokinetic parameters were extracted from medical records where available and included ADA levels before and after intensification and biomarkers including CRP and FC before and after intensification. The proportion of patients with ADA levels <5, between 5 and 10, and >10 µg mL^−1^ prior to and following treatment intensification was also determined. These cutoff values were selected based on ADA exposure–response relationship studies to reflect minimum and maximal clinically relevant levels.^22–26^

### Statistical Analysis

For categorical variables, percentages were calculated (with their 95% CIs). The descriptive analysis of quantitative variables calculated the mean and SD, or the median and interquartile range (IQR), depending on whether or not they had a normal distribution. The Wilcoxon signed-rank test was used to assess differences in FC, CRP, and ADA levels after intensification to ADA 80 mg eow. In the univariate analysis, categorical variables were compared using the chi-square test and quantitative variables using the appropriate test. Variables associated with clinical remission after intensification to ADA 80 mg eow were identified by logistic regression analysis showing the odds ratio (OR) and 95% CI and included time from diagnosis to start of ADA treatment (years), sex, type of IBD, smoking status, location and phenotype of CD (L and B), perianal disease, extension of UC, bionaive status, reason for intensification, concurrent use of immunomodulators, IBD activity before intensification, time from ADA initiation to intensification to 80 mg eow, drug levels before and after intensification to ADA 80 mg eow, endoscopy score before and after intensification to ADA 80 mg eow, and improvement of endoscopy and/or imaging tests after intensification to ADA 80 mg eow.

The level of statistical significance was set at 0.05 and the statistical package used was Stata v.15.1.

### Ethical Considerations

The study was approved by the Research Ethics Committee (CEIm) at Hospital Universitario Puerta de Hierro-Majadahonda, Madrid (Spain), prior to study initiation and a waiver for informed consent was granted.

The study complied with the provisions in the EU and Spanish legislation for Data Protection, the Spanish legislation for observational research with medicines and international recommendations for biomedical research, including the Declaration of Helsinki 2013.

## Results

**Figure 1.**
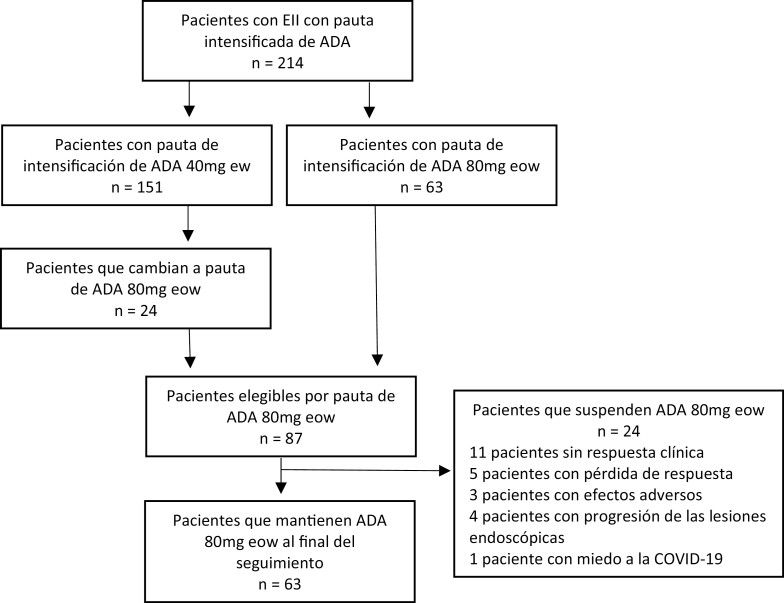
Flowchart of patient inclusion and follow-up. Abbreviations: ADA, adalimumab; eow, every other week; ew, every week.

A total of 87 patients (72 CD and 15 UC) were included (Figure 1). Baseline characteristics of all included patients are shown in Table 1.

**Table 1.** Baseline characteristics of patients who received adalimumab treatment intensification of 80 mg every other week.

Characteristic	Clinical remission achieved on intensification (*n* = 75)		Nonclinical remission on intensification (*n* = 12)		All (*n* = 87)	
Male sex, *n* (%)	46 (61)		5 (42)		51 (59)	
Median age, years (IQR)	50 (40–60)		50 (39–60)		50 (40–60)	
Type of IBD, *n* (%)						
CD	61 (81)		11 (92)		72 (83)	
UC	14 (19)		1 (8)		15 (17)	
Duration of illness prior to initiation of adalimumab, median years (IQR)	14 (7–22)		12 (5.5–14.5)		14 (7–22)	
Montreal classification—location, *n* (%)	*CD*	*UC*	*CD*	*UC*	*CD*	*UC*
	L1: 29 (48)	E1: 1 (7)	L1: 6 (55)	E2: 1 (100)	L1: 35 (49)	E1: 2 (13)
	L2: 10 (16)	E2: 6 (43)	L2: 4 (36)		L2: 14 (19)	E2: 6 (40)
	L3: 22 (36)	E3: 7 (50)	L3: 1 (9)		L3: 23 (32)	E3: 7 (47)
Montreal classification—phenotype, *n* (%)	*CD*	—	*CD*	—	*CD*	—
	B1: 31 (51)		B1: 7 (64)		B1: 38 (53)	
	B2: 20 (33)		B2: 2 (18)		B2: 22 (30)	
	B3: 10 (16)		B3: 2 (18)		B3: 12 (17)	
Perianal disease, *n* (%)	17 (28)	—	6 (50)	—	23 (32)	—
Immunosuppressant therapy at the time of ADA treatment intensification, *n* (%)						
Any	37 (49)		7 (58)		44 (51)	
Azathioprine	31 (84)		6 (86)		37 (84)	
Methotrexate	6 (16)		1 (14)		7 (16)	
Biological treatment prior to initiation of ADA, *n* (%)	54 (72)		7 (58)		61 (70)	
Indicators for adalimumab treatment, *n* (%)						
Corticodependence	22 (29)		2 (17)		24 (28)	
Perianal disease	6 (8)		3 (25)		9 (10)	
Treatment of postsurgical recurrence	11 (15)		3 (25)		14 (16)	
Prevention of postsurgical recurrence	5 (7)		0 (0)		5 (6)	
Severe active luminal disease	31 (41)		4 (33)		35 (40)	

Abbreviations: ADA, adalimumab; B1, inflammatory; B2, stenosing; B3, fistulizing; CD, Crohn’s disease; E1, colitis limited to the rectum; E2, left colitis; E3, extensive colitis; IBD, inflammatory bowel disease; IQR, interquartile range; L1, ileal; L2, colonic; L3, ileo-colonic; UC, ulcerative colitis.

The clinical factors precipitating ADA treatment intensification were ADA low levels—<5 µg mL^−1^—for 15 patients (17%), ADA low levels—<5 µg mL^−1^—and loss of response for 55 patients (63%), clinical response with failure to achieve clinical remission for 13 patients (15%), and active disease on objective evaluation (including colonoscopy, MRI, capsule endoscopy, and/or intestinal ultrasound) for 4 patients (5%). The proportion of patients achieving clinical remission according to the reason for intensification was as follows:

- ADA low levels for 15 patients (17%): 15/15 (100%).
- ADA low levels without clinical response for 55 patients (63%): 49/55 (90%).
- Clinical response without clinical remission for 13 patients (15%): 7/13 (54%).
- Active disease on objective evaluation (including colonoscopy, MRI, capsule endoscopy, and/or intestinal ultrasound) for 4 patients (5%): 4/4 (100%).

### Disease Activity

In all, 24 patients (28%) underwent dose escalation to 40 mg ew prior to the 80 mg eow intensification regimen. Of these, 9 patients (37%) had clinically active disease at the time of the change from the 40 mg ew regimen to the 80 mg eow regimen. Two patients discontinued treatment after a mean of 14.5 months (SD 16.3) after initiating ADA 80 mg eow. Both were in clinical remission following treatment intensification and subsequently discontinued treatment due to loss of response. Patient’s plasma ADA levels were >10 µg mL^−1^ prior to treatment intensification and <5 µg mL^−1^ at the time of treatment discontinuation.

**Figure 2.**
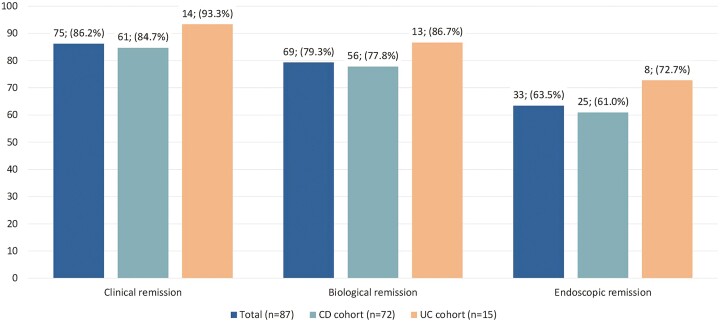
Clinical remission following adalimumab treatment intensification to 80 mg every other week. Following intensification to adalimumab 80 mg eow, patients were assessed at 1–2 months, thereafter every 3–6 months. Abbreviations: CD, Crohn’s disease; eow, every other week; IQR, interquartile range; UC, ulcerative colitis.

Following treatment intensification to ADA 80 mg eow, 75 patients (86%; 61 patients with CD and 14 patients with UC) were in clinical remission and 69 patients (79%) were in biologic remission (Figure 2). Univariate analysis indicated that none of the clinical factors evaluated were associated with attainment of clinical remission consequently multivariate analyses were not performed (Table 2).

**Table 2.** Univariate analysis of variables associated with clinical, biological, and endoscopic remission.

	*n*	Remission	No remission	Univariate analysis RR (IC 95%); *P*
Clinical remission (*n* = 87)				
Total, *n* (%)		75 (86)	12 (14)	
Sex, *n* (%)				
Male	51	46 (61)	5 (42)	2.21 (0.64–7.66); .207
Female (Ref)	36	29 (39)	7 (58)	
Age, mean (SD)	87	49 (14.3)	49 (14.3)	.953
Location EC, *n* (%)				
Ileal (Ref)	35	29 (48)	6 (55)	
Colonic	14	10 (16)	4 (36)	0.52 (0.12–2.22); .375
Ileocolonic	23	22 (39)	1 (9)	4.55 (0.51–40.6); .175
Location CU, *n* (%)				
Proctitis (Ref)	2	1 (7)	1 (100)	
Left	6	6 (43)	0	.047
Extensive	7	7 (50)	0	.047
Phenotype (EC), *n* (%)				
Inflammatory (Ref)	38	31 (51)	7 (64)	
Stenosing	22	20 (33)	2 (18)	2.26 (0.43–11.98); .329
Penetrating	12	10 (16)	2 (18)	1.13 (0.20–6.34); .890
Naive to biologics, *n* (%)				
Yes	61	54 (72)	7 (58)	1.84 (0.52–6.43); .337
No (Ref)	26	21 (28)	5 (42)	
Concurrent ISS, *n* (%)				
Yes	44	37 (49)	7 (58)	0.70 (0.20–2.39); .563
No (Ref)	43	38 (51)	5 (42	
Years of evolution until the start of ADA 80 mg eow, mean (SD)	87	2 (2.9)	0.9 (1.7)	.822
Calprotectin prior to ADA 80 mg eow (µg g^−1^), mean (SD)	87	546 (1093)	835 (920)	.390
CRP prior to ADA 80 mg eow (mg dL^−1^), mean (SD)	87	8.5 (13.1)	9.5 (8.2)	.808
Endoscopic score prior to ADA 80 mg eow, mean (SD)	75	1.7 (0.8)	2.2 (0.9)	.075
Biologic remission (*n* = 87)				
Total, *n* (%)		69 (79)	18 (21)	
Sex, *n* (%)				
Male	51	42 (61)	9 (50)	1.56 (0.55–4.41); .404
Female (Ref)	36	27 (39)	9 (50)	
Age, mean (SD)	87	49 (14.2)	48 (16.9)	.890
Location EC, *n* (%)				
Ileal (Ref)	35	25 (45)	10 (63)	
Colonic	14	10 (18)	4 (25)	1 (0.25–3.94); 1
Ileocolonic	23	21 (37)	2 (12)	4.20 (0.83–21.33); .068
Location CU, *n* (%)				
Proctitis (Ref)	2	1 (8)	0 (0)	
Left	6	5 (38)	1 (50)	5 (0.15–166.59); .346
Extensive	7	7 (54)	1 (50)	.047
Phenotype (EC), *n* (%)				
Inflammatory (Ref)	38	30 (54)	8 (50)	
Stenosing	22	19 (34)	3 (19)	1.69 (0.49–7.17); .477
Penetrating	12	7 (12)	5 (31)	0.37 (0.09–1.49); .156
Naive to biologics, *n* (%)				
Yes	61	49 (71)	12 (67)	1.22 (0.40–3.71); .720
No (Ref)	26	20 (29)	6 (33)	
Concurrent ISS, *n* (%)				
Yes	44	33 (48)	11 (39)	0.56 (0.20–1.68); .315
No (Ref)	43	36 (52)	7 (61)	
Years of evolution until the start of ADA 80 mg eow, mean (SD)	87	2.8 (2.9)	1.3 (2.1)	.784
Calprotectin prior to ADA 80 mg eow (µg g^−1^), mean (SD)	87	548 (1130)	732 (812)	.518
CRP prior to ADA 80 mg eow (mg dL^−1^), mean (SD)	87	8.4 (13.5)	9.4 (7.9)	.768
Endoscopic score prior to ADA 80 mg eow, mean (SD)	75	1.7 (0.8)	2.1 (0.9)	.053
Endoscopic remission (*n* = 48)				
Total, *n* (%)		27 (56)	21 (44)	
Sex, *n* (%)				
Male	25	15 (56)	10 (48)	
Female (Ref)	23	12 (44)	11 (52)	0.77 (0.25–2.41); .657
Age, mean (SD)	48	49 (13.9)	50 (16.6)	.530
Location EC, *n* (%)				
Ileal (Ref)	18	9 (47)	9 (50)	
Colonic	7	3 (16)	4 (22)	1.73 (0.27–10.97); .558
Ileocolonic	12	7 (37)	5 (28)	0.97 (0.23–4.04); .966
Location UC, *n* (%)				
Proctitis (Ref)	1	1 (12)	0 (0)	
Left	3	2 (25)	1 (33)	0.50 (0.12–1.99); .248
Extensive	7	5 (63)	2 (67)	2.50 (0.10–62.6); .571
Phenotype (CD), *n* (%)				
Inflammatory (Ref)	22	12 (63)	10 (55)	
Stenosing	10	5 (26)	5 (28)	0.35 (0.08–1.53); .157
Penetrating	5	2 (11)	3 (17)	0.18 (0.02–1.22); .063
Naive to biologics, *n* (%)				
Yes	37	21 (78)	16 (76)	0.99 (0.25–3.95); .986
No (Ref)	11	6 (22)	5 (24)	
Concurrent ISS, *n* (%)				
Yes	23	11 (41)	12 (57)	0.61 (0.19–1.89); .388
No (Ref)	25	16 (59)	9 (43)	
Years of evolution until the start of ADA 80 mg eow, mean (SD)	48	2.1 (2.1)	2.9 (3.1)	.314
Calprotectin prior to ADA 80 mg eow (µg g^−1^), mean (SD)	48	434 (780)	784 (1468)	.376
CRP prior to ADA 80 mg eow (mg dL^−1^), mean (SD)	48	8.6 (13.1)	7.9 (13.4)	.151
Endoscopic score prior to ADA 80 mg eow, mean (SD)	48	0.07 (0.27)	1.86 (0.77)	.125

Abbreviations: ADA, adalimumab; CD, Crohn’s disease; CRP, C-reactive protein; eow, every other week; IBD, inflammatory bowel disease; ISS, immunosuppressant; UC, ulcerative colitis.

The proportion of patients with objective measures of disease activity prior to and following treatment intensification are shown in Table 3. Prior to intensification, objective assessment of activity was available for 79 patients (91%) and for 52 patients (60%) following intensification. Among the 79 patients with objective assessment of activity prior to intensification, 68 (86%) had active disease. Endoscopic evaluation results were available both prior to and following ADA intensification to 80 mg eow for 48 patients (55%). The mean endoscopic disease activity score prior to ADA intensification was 1.8 (SD 0.75). Among this subset of patients, 27 (56%) demonstrated an improvement, 17 (35%) demonstrated no change, and 4 (9%) experienced worsening. The mean endoscopic disease activity score following ADA intensification was 0.6 (SD 1.01).

**Table 3.** Proportion of patients with objective assessment prior to and following intensification to adalimumab 80 mg every other week.

Assessment, *n* (%)	Prior to intensification	Following intensification^a^
Any objective assessment of activity	79 (91)	52 (60)
Ileocolonoscopy	75 (95)	48 (92)
Endoscopic capsule	0 (0)	1 (2)
MRI	3 (4)	1 (2)
Intestinal ultrasound	1 (1)	2 (4)

Abbreviations: eow, every other week; IQR, interquartile range; MRI, magnetic resonance imaging.

^a^Median time between objective assessment prior to escalation and escalation to adalimumab 80 mg eow: median 3.5 (IQR 1–10) months. Time between intensification to adalimumab 80 mg eow and objective assessment: median 8 (IQR 5–13) months.

### Pharmacokinetics

Prior to treatment intensification, the median ADA levels were 4.2 µg mL^−1^ (IQR 1.9–8.6). At this timepoint, 50 patients (57%) had ADA levels <5 µg mL^−1^, 19 patients (22%) had ADA levels between 5 and 10 µg mL^−1^, and 18 patients (21%) had ADA levels >10 µg mL^−1^. Following treatment intensification, the median ADA levels were 8.5 µg mL^−1^ (IQR 6.4–13). At this timepoint, 15 patients (17%) had ADA levels <5 µg mL^−1^, 40 patients (46%) had ADA levels between 5 and 10 µg mL^−1^, and 32 patients (37%) had ADA levels >10 µg mL^−1^. The median ADA level among patients remaining on treatment at a median of 14 months (IQR 7–20 months) after treatment intensification was 10.7 µg mL^−1^ (IQR 7.7–15.0). For this group of patients, 35 (55%), 13 (21%), and 15 (24%) patients had ADA levels <5, between 5 and 10, and >10 µg mL^−1^, respectively, prior to treatment intensification. Following treatment intensification 6 (9%), 34 (54%), and 23 (37%) patients had ADA levels <5, between 5 and 10, and >10 µg mL^−1^, respectively (median time to first postintensification assessment 2 months [IQR 1–4]). For those patients who discontinued treatment (*n* = 24), the median ADA level at the last assessment prior to discontinuation was 5.8 µg mL^−1^ (IQR 0.6–9.5). For this group of patients, 15 (63%), 6 (25%), and 3 (12%) patients had ADA levels <5, between 5 and 10, and >10 µg mL^−1^, respectively, prior to treatment intensification. Following treatment intensification 9 (37.5%), 6 (25%), and 9 (37.5%) patients had ADA levels <5 µg mL^−1^, between 5 and 10.0, and >10 µg mL^−1^, respectively (median time to first postintensification assessment 2 months [IQR 1–4]).

**Figure 3.**
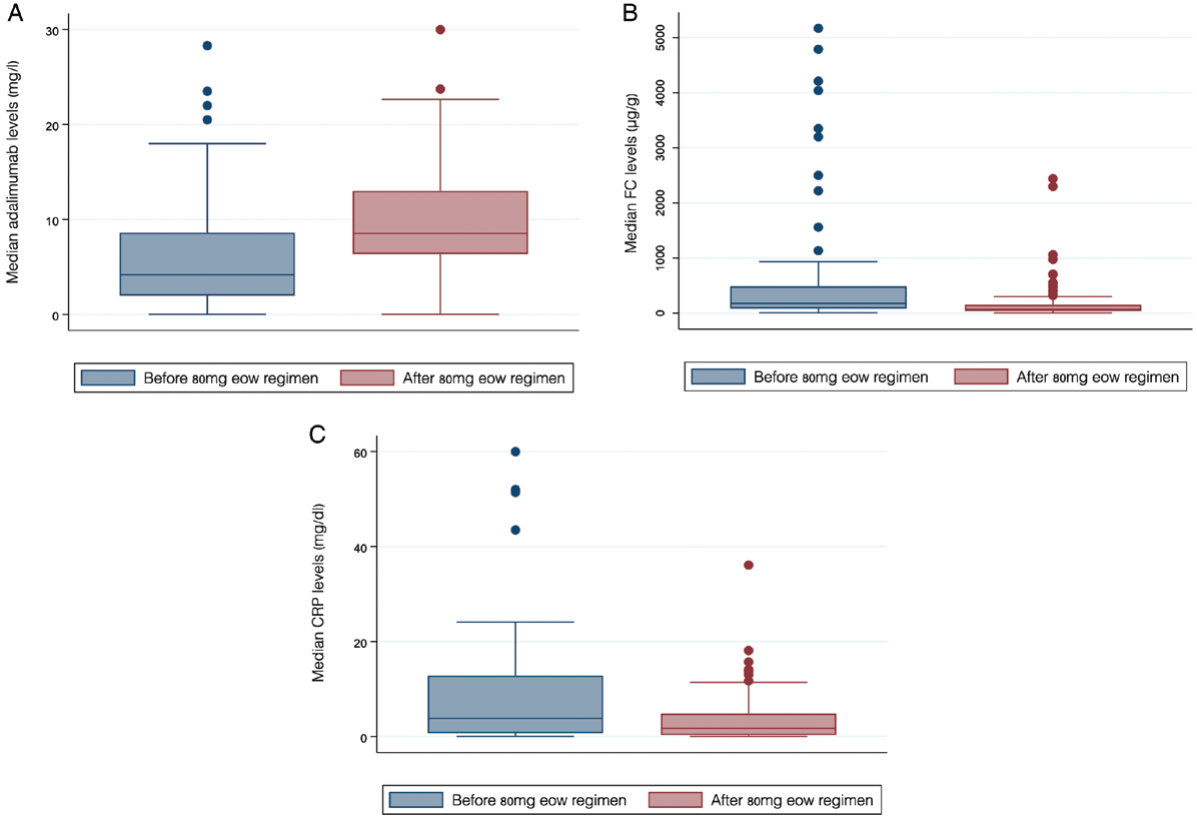
Change from baseline in (A) adalimumab levels, (B) fecal calprotectin, and (C) C-reactive protein levels from baseline following treatment intensification to an 80 mg every other week regimen. Abbreviations: CRP, C-reactive protein; eow, every other week; FC, fecal calprotectin.

Biochemical parameters (FC and CRP) prior to and a median of 5 months (IQR 4–6 months) following treatment intensification to ADA 80 mg eow are shown in Table 4. Changes in FC, CRP, and ADA levels from the prior ADA regimen were statistically significant (*P* < .001) (Figure 3).

**Table 4.** Pharmacokinetic parameters prior to and following intensification to adalimumab 80 mg every other week after a median of 5 months follow-up (IQR 4–6 months).

Parameter	Prior to intensification	Following intensification
ADA (µg mL^−1^), median (IQR)	4.2 (1.9–8.6)	8.5 (6.4–13)
FC (µg g^−1^), median (IQR)	176 (83–485)	70 (39–150)
CRP (mg mL^−1^), median (IQR)	3.8 (0.7–12.8)	1.7 (0.3–4.8)

Abbreviations: ADA, adalimumab; CRP, C-reactive protein; FC, fecal calprotectin; IQR, interquartile range.

### Treatment Discontinuation

**Figure 4.**
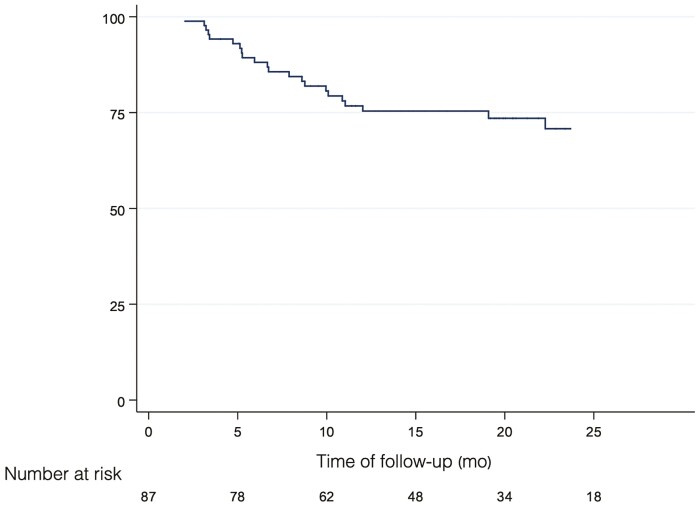
Proportion of patients remaining on treatment and in clinical remission with adalimumab 80 mg every other week. Abbreviation: mo, months.

A total of 24 patients (28%) discontinued treatment after a median of 6.5 months (IQR 4.5–10.5). Lack of clinical response was given as the reason for treatment discontinuation for 11 patients (46%), loss of response for 5 patients (21%), adverse events for 3 patients (12%; psoriasis), progression of endoscopic lesions for 4 patients (17%), and patient’s own decision due to concerns about COVID-19 for 1 patient (4%). After a median follow-up of 19 months (IQR 13–25) 63 patients (72%) remained on treatment and in clinical remission (Figure 4). The 3 patients who discontinued treatment due to the emergence of an adverse event of psoriasis all responded well to discontinuation and to alternative therapy with ustekinumab. There were no serious infections, hospitalizations, or deaths in the ADA 80 mg eow regimen.

### Predictors of Treatment Discontinuation

In multivariate analysis, being in clinical remission at the time of treatment intensification reduced the risk of treatment discontinuation by 83% (hazard ratio [HR] 0.17, 95% CI 0.02–1.26, *P* = .02), having achieved biological remission reduced the risk by 96.8% (HR 0.03, 95% CI 0.01–0.09, *P* < .001), having ADA levels ≥5 µg mL^−1^ after intensification 80 mg eow reduced the risk by 48% (HR 0.52, 95% CI 0.3–0.92, *P* = .02), and having ADA levels ≥5 µg mL^−1^ before intensification 80 mg eow reduced the risk by 77% (HR 0.23, 95% CI 0.1–0.54) (Table 5).

**Table 5.** Factors predictive of discontinuation of treatment with adalimumab 80 mg every other week.

Variable	HR	95% CI	*P*
Male (vs female)	0.6	0.27–1.34	.21
Crohn’s disease (vs ulcerative colitis)	0.58	0.17–1.96	.35
Smokers (vs nonsmokers)	1.56	0.95–2.59	.08
Years to start of ADA 80 mg eow	0.96	0.91–1.01	.07
Combination with ISS (vs monotherapy)	1.17	0.52–2.62	.69
Indication for ADA	1.26	0.98–1.61	.06
Bionaive (vs non bionaive)	0.7	0.31–1.61	.41
Clinical remission at time of ADA 80 mg eow (vs non clinical remission)	1.17	0.02–1.26	.02
Biological remission at the time of initiation of ADA 80 mg eow (vs non biological remission)	0.03	0.01–0.09	<.001
ADA levels ≥5 µg mL^−1^ after intensification 80 mg eow (vs ADA levels <5 µg mL^−1^)	0.52	0.3–0.92	.02
ADA levels ≥5 µg mL^−1^ before intensification 80 mg eow (vs ADA levels <5 µg mL^−1^)	0.23	0.1–0.54	.03
Calprotectin before the start of ADA 80 mg eow (µg g^−1^)	1.01	0.99–1.01	.64
CRP before the start of ADA 80 mg eow (mg dL^−1^)	1.06	0.98–1.03	.66
Endoscopic activity before the start of ADA 80 mg eow moderate to severe (vs mild)	1.38	0.5–3.8	.52

Abbreviations: ADA, adalimumab; CRP, C-reactive protein; eow, every other week; HR, hazard ratio; ISS, immunosuppressant.

### Economic Evaluation

At our center the cost of 2 × 40 mg dose pens and one 80 mg dose pen were equivalent. The cost of treatment did not increase with the 80 mg eow regimen compared with the standard 40 mg eow regimen.

## Discussion

The availability of biological therapy has transformed the treatment of IBDs such as UC and CD with improved symptom control, mucosal healing, and improved long-term outcomes compared with standard pharmacological agents. These agents have enabled a change in the treatment paradigm from symptom control to prevention of disease progression.^27^ Optimization of biological therapy regimens is emerging as an important component of the management strategy as a proportion of patients will not respond to their initial regimen, will lose an initial clinical response over time or will fail to achieve complete symptom control (clinical remission).^27,28^ For these patients, switching treatment or intensification of their current regimen are options. The requirement for treatment intensification among patients with IBD receiving first-line anti-TNFα therapies is common, with estimates indicating around one-third of such patients will require treatment intensification.^28^ High rates of treatment discontinuation due to lack of appropriate disease control, including loss of response, indicate the need for appropriate intensification regimens.^29,30^ The approval of ADA administered as 80 mg eow for the treatment of patients with IBD as an alternative to the standard intensification regimen of 40 mg ew offers an alternative intensification regimen that some patients may find more convenient. In this retrospective analysis, treatment intensification to ADA 80 mg eow among patients with IBD receiving treatment with ADA 40 mg ew was associated with high rates of both clinical and biological remission.

Therapeutic drug monitoring for patients with IBD receiving anti-TNFα therapy is increasingly recognized as an important component of the ongoing management of such patients.^7^ Current guidelines recommend switching to an alternative biologic agent in case of treatment failure.^1,2^ However, treatment failure (loss of response, failure to achieve clinical remission, or failure to achieve therapeutic goals associated with improved outcome and quality of life) or loss of response may be a consequence of subtherapeutic drug plasma levels.^31^ Indeed, recent data suggest that subtherapeutic drug levels of biological agents may be associated with the development of antidrug antibodies, a situation shown to further reduce plasma drug levels and negatively impact response.^32,33^ In this case, intensification of treatment may be an efficacious approach to ensure therapeutic drug plasma levels and achieve and maintain optimal response and disease control. To identify patients for ADA treatment intensification we utilized an ADA level of <5 µg mL^−1^ as an appropriate indicator of subtherapeutic plasma levels. Given the heterogeneity of current recommendations for appropriate cutoffs and the observation that higher ADA levels may be associated with mucosal healing we also utilized cutoffs of 5–10 and >10 µg mL^−1^. These cutoff values were selected based on recent data on recommended therapeutic ADA levels to achieve clinical and biologic remission and mucosal healing.^22–26^ We observed a reduction in the proportion of patients with subtherapeutic ADA plasma levels <5 µg mL^−1^ and an increase in the proportion of patients with ADA plasma levels 5–10 and >10 µg mL^−1^, levels associated with mucosal healing,^25,26^ following treatment intensification. These data, combined with the observation that 75 patients (86%; 61 patients with CD and 14 patients with UC) were in clinical remission and 69 patients (79.3%) were in biologic remission following treatment intensification, suggest that a proactive approach utilizing therapeutic drug monitoring, may be appropriate to both achieve and deepen the clinical response in these patients.^34^ Indeed, therapeutic drug monitoring and treatment intensification may also be of value for particular clinical challenges such as persistent perianal fistulas. A recent cross-sectional study of patients with CD-associated perianal fistulas treated with ADA revealed a significant association between high serum ADA levels and clinical remission of fistulas.^34^ A number of questions and concerns remain to be addressed with regard to therapeutic drug monitoring of biological therapy in IBD. These include whether a reactive (on failure to respond or loss of response) or proactive (for all patients at least once during maintenance therapy) is appropriate and alignment of plasma level cutoffs with therapeutic goals.^24^

No less important than clinical remission rates with respect to treatment optimization for patients with IBD is the acceptability of the intensified regimen. The 40 mg ew intensification regimen utilizes an increased frequency of administration. Recent studies of patient satisfaction with ADA treatment have revealed a preference among patients for an ADA 80 mg eow range versus the standard 40 mg ew regimen.^35^ Although a formal evaluation of safety and tolerability was not undertaken, the observation that an adverse event (psoriasis) was the reason for treatment discontinuation of 3 patients (12%) and that no serious infections, hospitalizations, or deaths in the ADA 80 mg eow regimen is reassuring and consistent with the already known safety profile of ADA.^14,15^

Limitations of the current study were the observational nature of the data collection and lack of a comparator arm to anchor the observed results to alternative treatments or treatment regimens. In addition, the single-center design and relatively small number of patients included in the study likely limited the statistical power to reveal any differentiating factors between those achieving and not achieving clinical remission. Indeed, the univariate analysis did not identify an association between any of the clinical factors evaluated and the attainment of clinical remission. Despite these limitations, the current study provides an insight into a novel treatment strategy in real-world clinical practice.

## Conclusions

The results of this retrospective analysis showed that intensification of ADA therapy to 80 mg eow in patients with IBD was safe, effective, and did not increase drug costs at our center with respect to standard intensification to 40 mg ew.

- What is already known? Inadequate or loss of response to adalimumab in patients with inflammatory bowel disease can be managed by intensification of treatment to 40 mg every week.
- What is new here? Treatment intensification to an alternative regimen of adalimumab 80 mg every other week was safe and effective in a real-world setting with high rates of clinical and biologic remission and no serious infections, hospitalizations, or deaths.
- How can this study help patient care? These results provide confidence that this alternative intensification regimen is safe and effective for patients with IBD experiencing inadequate or loss of response to adalimumab.

## Data Availability

Data not publicly available.
